# Childhood cause-specific mortality in rural Western Kenya: application of the InterVA-4 model

**DOI:** 10.3402/gha.v7.25581

**Published:** 2014-10-29

**Authors:** Nyaguara O. Amek, Frank O. Odhiambo, Sammy Khagayi, Hellen Moige, Gordon Orwa, Mary J. Hamel, Annemieke Van Eijk, John Vulule, Laurence Slutsker, Kayla F. Laserson

**Affiliations:** 1 Center for Global Health Research, Kenya Medical Research Institute, Kisumu, Kenya; 2 Center for Global Health, Centers for Disease Control and Prevention, Atlanta, GA, USA

**Keywords:** under-5, verbal autopsy, InterVA, health and demographic surveillance

## Abstract

**Background:**

Assessing the progress in achieving the United Nation's Millennium Development Goals in terms of population health requires consistent and reliable information on cause-specific mortality, which is often rare in resource-constrained countries. Health and demographic surveillance systems (HDSS) have largely used medical personnel to review and assign likely causes of death based on the information gathered from standardized verbal autopsy (VA) forms. However, this approach is expensive and time consuming, and it may lead to biased results based on the knowledge and experience of individual clinicians. We assessed the cause-specific mortality for children under 5 years old (under-5 deaths) in Siaya County, obtained from a computer-based probabilistic model (InterVA-4).

**Design:**

Successfully completed VA interviews for under-5 deaths conducted between January 2003 and December 2010 in the Kenya Medical Research Institute/US Centers for Disease Control and Prevention HDSS were extracted from the VA database and processed using the InterVA-4 (version 4.02) model for interpretation. Cause-specific mortality fractions were then generated from the causes of death produced by the model.

**Results:**

A total of 84.33% (6,621) childhood deaths had completed VA data during the study period. Children aged 1–4 years constituted 48.53% of all cases, and 42.50% were from infants. A single cause of death was assigned to 89.18% (5,940) of cases, 8.35% (556) of cases were assigned two causes, and 2.10% (140) were assigned ‘indeterminate’ as cause of death by the InterVA-4 model. Overall, malaria (28.20%) was the leading cause of death, followed by acute respiratory infection including pneumonia (25.10%), in under-5 children over the study period. But in the first 5 years of the study period, acute respiratory infection including pneumonia was the main cause of death, followed by malaria. Similar trends were also reported in infants (29 days–11 months) and children aged 1–4 years.

**Conclusions:**

Under-5 cause-specific mortality obtained using the InterVA-4 model is consistent with existing knowledge on the burden of childhood diseases in rural western Kenya.

Monitoring the progress of the United Nation's Millennium Development Goals in terms of population health requires consistent and reliable information on cause-specific mortality patterns. In addition, information about the distribution of causes of death is crucial for public health planning, resource allocation, and evaluating the impact of interventions. In many developing countries, where the highest burden of childhood mortality is found, this information is often missing or inaccurate due to weak health systems, poor attendance at health facilities, weak civil registration and death certification systems, as well as the fact that most deaths occur at home (1, 2).

In the recent past, the existence of health and demographic surveillance systems (HDSS) in various parts of low– and middle-resource countries has provided invaluable field data on all-cause and cause-specific mortality patterns in such populations (1, 3–5). Typically, HDSS tracks a limited and common set of key variables determining population dynamics and demographic trends in a geographically defined population through routine collection of information on births, deaths, and migrations. In addition, most of the HDSS sites collect information on health outcomes (e.g. causes of death using verbal autopsy (VA), incidence, and/or the prevalence of particular diseases of public importance) (6).

VA techniques entail interviewing the main caregiver (in most cases, a relative) of the recently deceased individual to gather information on the circumstances surrounding the death (5, 7–10). It is assumed that the respondent would be able to recognize and recall premorbid signs and symptoms and would volunteer such information during the VA interview. The information obtained is then interpreted to derive the most probable cause of death. VA interviews are conducted by trained field workers using structured VA questionnaires. The questionnaire consists of a narrative section for recording a verbatim account of the circumstances leading to death and a close-ended section with filter questions on signs and symptoms of disease and/or injury (11). Until recently, various HDSS sites have been using diverse VA tools. A standardized VA tool has been proposed by the World Health Organization (WHO) in collaboration with the INDEPTH Network and other partners (12).

Methods to interpret collected VA data to determine the most probable cause of death have varied over the years. Coding by medical personnel is the most commonly used method and typically involves the independent review of the data by at most three clinicians. These clinicians review each completed VA questionnaire and assign a most probable cause of death using the International Classification of Disease version 10 (ICD-10) lists or an abridged version (13, 14). A number of challenges have been associated with this method (medical personnel reviews) despite studies (15, 16) that validate it. The review process demands a considerable amount of clinicians’ time and may be costly, particularly if the initial level of agreement between clinicians is poor. The clinicians may also differ systematically in interpreting VA data based on their experience and/or exposure to the local epidemiology of diseases, which may lead to inconsistencies in reporting on the cause of death.

Consequently, computer-based methods such as expert/data-driven algorithms, neural networks, and probabilistic models such as InterVA have been proposed as alternative methods of obtaining the cause of death. However, only the InterVA method has been explored in a number of settings (7, 10, 17–20). The remaining methods are not readily available. Furthermore, most of these (7, 10, 17–20) studies have focused on adults, and little is known about under-5 cause-specific mortality. InterVA is based on the Bayes’ theorem, and it is an open-source program (21), which requires less labor, time, and cost compared to clinician-based methods.

In this article, we describe childhood cause-specific mortality trends based on results obtained from the InterVA version 4 (InterVA-4) model in interpreting childhood VA data collected by the Kenya Medical Research Institute (KEMRI) in collaboration with the US Centers for Disease Control and Prevention (CDC) from the KEMRI/CDC HDSS from 2003 to 2010.

## Methods

### Study area and population

The characteristics and profile of the KEMRI/CDC HDSS have been described in detail elsewhere (6). In brief, KEMRI/CDC HDSS was launched in September 2001 by the CDC in collaboration with KEMRI in the Asembo area of Siaya County; it expanded to the Gem area in 2003, then to the Karemo area in 2007 in the same county in rural western Kenya. The data used in this study were from the Gem and Asembo areas, which cover about 500 km^2^ and have a population of approximately 150,000 residing in 216 villages; under-5 children account for about 15% of the total population (22). The residents of the study area are predominantly Dholuo speaking, and they derive their livelihood mainly from subsistence farming. The HDSS area is both malaria and HIV endemic, with a prevalence of 34% in children less than 5 years and 13 % in adults respectively (6).

### Data collection

The HDSS collects core demographic events such as births, deaths, and migrations three times a year. Trained field workers visit every household and interview an appropriate respondent who is available at the time of the visit. Individual information is checked for every member of the house, and all events that have occurred since the previous census are recorded. Residents of the study area tend to underreport neonatal (<28 days) deaths, particularly if they occur within the first week of delivery. Therefore, to increase the chances of capturing all neonatal deaths, we engage at least one village reporter (who is a resident) in each village for the notification of deaths and births as they occur. This information is then merged into the HDSS database after verification. The HDSS uses the VA method to ascertain the cause of death for all deaths that occur within the study area.

### Verbal autopsy

The VA procedure used in this study has been described elsewhere in detail (5). In brief, VA questionnaires are processed using the deceased's demographic information obtained from the deaths notified by village reporters. The information includes name, age, parental details, and the location where and date when the death occurred to help the VA interviewer identify the deceased's residential location for interview. VA interviews are conducted by trained workers (holding a minimum qualification of a Kenyan secondary school certificate) at least 3 weeks from the date of death to allow for the mourning period to elapse. In cases where an appropriate respondent is not identified during the first visit, the interviewer schedules two more visits before declaring the interview not done (‘no appropriate respondent’). It takes approximately 30–45 min to administer the VA questionnaire. Regular refresher training on VA data collection is given to VA interviewers at least three times a year, and all the collected data are subjected to logical checks to ensure compatibility with the demographic information and the skip patterns in line with the questionnaire.

During the study period, the VA questionnaire has changed three times: From 2003 to 2007, we adopted the VA questionnaire developed by INDEPTH HDSS sites around the world with modifications tailored to the study area (5). From 2008 to May 2009, we switched to the Sample Vital Registration with Verbal Autopsy (SAVVY) tool (8), and since then we have been using the WHO 2007 questionnaire (23).

### The InterVA model

The InterVA model is an expert opinion–based algorithm founded on Bayes’ theorem, which defines the probability of a cause of death given the presence of a particular symptom or set of symptoms (indicators). This model has been discussed extensively in a number of studies (17, 21, 24, 25). In brief, the model gives at most three possible causes of death for each case with their corresponding likelihood values. The first cause represents the primary cause of death. The local population-based prevalence of malaria and HIV/AIDS is input into the model. For this study, we used InterVA-4 (version 4.02) (24) and specified high malaria and high HIV/AIDS prevalence conditions.

### Data analysis

Due to differences in the three questionnaires, a program was written in STATA (version 11.0) to extract as many as possible of the 2012 WHO InterVA indicators (23) for each VA record of residents from 2003 to 2010 who were younger than 5 years old at the time of death. Pediatric residents of the HDSS are defined as children who have lived in the HDSS area for at least four continuous calendar months (6). VA records that did not contain any symptom data were excluded. The resulting data set from this script was then processed using InterVA-4 (version 4.02), and the cause of death summarized into cause-specific mortality fractions (CSMF), taking into account all three possible causes per case. Deaths were grouped into three age categories: neonates (≤28 days), infants (1–11 months), and children (1–4 years), and in each age group, the cause-specific mortality rate was calculated as the number of deaths per 1,000 person-years at risk (pyrs), except for neonates in which the rate was per 1,000 live births. This data set is also part of a multisite cause-specific mortality database (26).

### Ethical consideration

Informed written consent was obtained from the compound heads for participation of their households. The HDSS activities, of which our study is a part, were reviewed and approved by the institutional review boards of both CDC (Atlanta, GA) and KEMRI (Nairobi, Kenya).

## Results

**Fig. 1:**
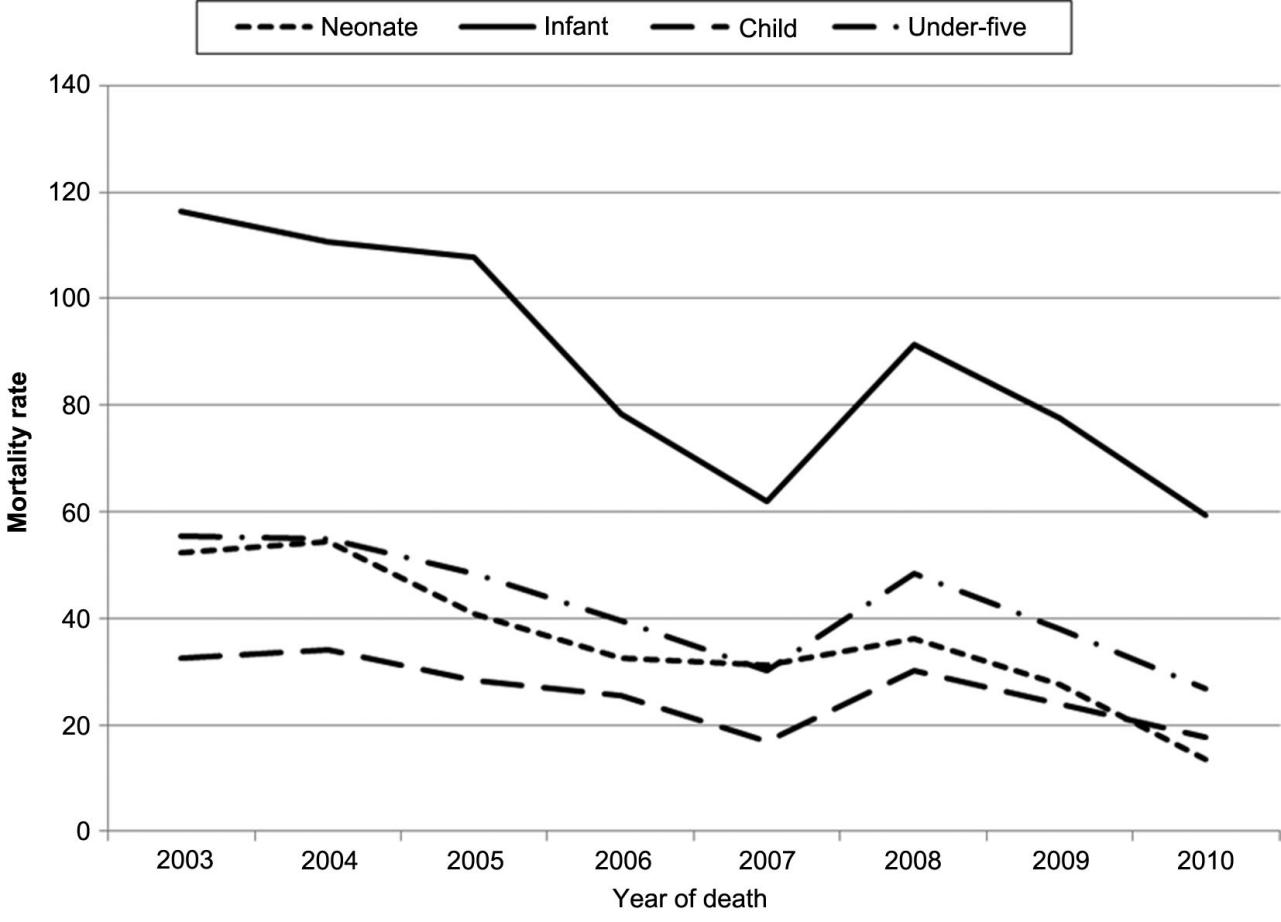
All-cause childhood mortality rate.

During the study period, 7,847 under-5 deaths with 184,338 pyrs were recorded in the KEMRI/CDC HDSS (Gem and Asembo areas). Most of these deaths occurred in children aged 1–4 years (47.85%), followed by infants (41.10%). Of all the childhood deaths, 51.11% were from males. However, female deaths in the infant age group (52.21%) were more compared to males in 2004. Figure 1 shows all-cause mortality rates per 1,000 pyrs except for neonatal rate (which was per 1,000 live births). Overall, the under-5 mortality rate declined by 8% (incidence rate ratio (IRR)=0.92, 95% confidence interval (CI: 0.91, 0.94) during the study period. Similar trends were observed in age-specific analysis. For instance, there was a 13% (IRR=0.87, 95% CI: 0.85, 0.90) decrease in neonates. Overall, males had a slightly higher mortality rate compared to females, although this was not significant (IRR=1.02, 95% CI: 0.98, 1.07).

Out of the total under-5 deaths, 84.33% (6,621) contained at least one symptom datum; of these, 48.53% of these cases occurred in children aged 1–4 years, while 42.50% were infant deaths. The InterVA-4 model assigned a single or primary cause of death in 5,940 (89.18%) cases, two causes of death in 556 (8.35%) cases, and three causes in 25 (0.38%) cases. In 140 (2.10%) cases, the model assigned the cause of death as ‘indeterminate’.

During the entire study period, malaria (29.29%) was the leading cause of death, followed by acute respiratory infection including pneumonia (27.88%), in under-5 children. However, analysis by year shows that in the first 5 years of the study period, acute respiratory infection including pneumonia was the main cause of death, followed by malaria. In the last 3 years of the study period, malaria was the main cause of death, then HIV/AIDS-related disease, with pneumonia being third (Supplementary Table 1).

Among neonates, birth asphyxia (34.68%) was the leading cause of death, followed by pneumonia (30.47%). Table 1 shows the CSMFs for neonatal deaths. Birth asphyxia increased from 37.65 to 47.46% in the first 5 years of the study period except in the year 2005, and it thereafter declined to 11.43% in 2010. In the same period, neonatal pneumonia cases decreased from 42.35 to 20.34%, then increased to 42.86% at the end of the study period. Neonatal deaths due to preterm birth steadily increased from 1.18 to 6.15% from 2003 to 2006, followed by a decline to 1.69% in 2007, then another steady increase to 11.43% in 2010. Congenital malformation deaths in neonates were observed in only the last 3 years of the study period.

**Table 1:** Cause-specific mortality fractions for neonates, by year

	Neonate							
	
	Calendar year							
	
	2003	2004	2005	2006	2007	2008	2009	2010
Number of deaths	85	83	65	65	59	120	82	35
WHO 2012 cause-of-death code								
10.02 Birth asphyxia	37.65	43.37	33.85	41.54	47.46	30.00	25.61	11.43
10.03 Neonatal pneumonia	42.35	33.73	33.85	29.23	20.34	20.83	29.27	42.86
10.99 Other and unspecified neonatal causes of death	12.94	14.46	21.54	16.92	22.03	20.83	17.07	8.57
10.04 Neonatal sepsis	4.71	4.82	4.62	4.62	6.78	13.33	9.76	14.29
01.07 Meningitis and encephalitis	1.18	1.20	–	–	1.69	4.17	1.22	5.71
10.01 Prematurity	1.18	1.20	4.62	6.15	1.69	4.17	8.54	11.43
12.05 Accident exposure to smoke, fire & flame	–	–	–	1.54	–	–	–	–
10.06 Congenital malformation	–	–	–	–	–	2.50	1.22	2.86
99 Indeterminate	–	1.20	1.54	–	–	4.17	7.32	2.86

WHO=World Health Organization.

Evaluation of the CSMFs for infants (see Table 2) suggests that acute respiratory infection including pneumonia was the leading cause of death (60.67%) in infants, followed by malaria (19.11%) in 2003. In 2010, by contrast, malaria was the main cause of death (36.49%). Infant mortality due to diarrheal diseases significantly increased over the first 4 years of the study period. An increase was also observed in meningitis and encephalitis in the last 3 years.

**Table 2:** Cause-specific mortality fractions for infants, by year

	Infant (29 days–11 months)							
	
	Calendar year							
	
	2003	2004	2005	2006	2007	2008	2009	2010
Number of deaths	450	429	368	330	279	441	306	211
WHO 2012 cause of death code								
01.02 Acute respiratory infection, including pneumonia	60.67	55.24	50.27	50.91	47.67	34.01	34.31	27.49
01.05 Malaria	19.11	24.01	22.28	18.48	17.20	32.65	35.62	36.49
01.03 HIV/AIDS-related death	12.22	7.23	11.96	11.52	14.70	8.39	12.09	13.74
01.04 Diarrheal diseases	3.56	6.99	8.97	11.52	10.75	11.79	7.52	9.95
01.06 Measles	1.56	3.50	2.17	3.03	1.79	1.59	2.61	1.90
01.10 Pertussis	0.22	–	–	–	–	–	–	–
01.07 Meningitis and encephalitis	0.22	0.23	–	0.61	0.36	1.13	1.63	3.79
01.01 Sepsis (non-obstetric)	–	0.47	0.27	–	0.72	0.23	0.33	–
04.03 Sickle cell with crisis	0.22	–	0.27	–	1.79	0.68	0.65	–
03.02 Severe malnutrition	0.44	–	–	0.91	0.36	0.68	0.33	1.42
08.01 Epilepsy	0.44	0.23	–	–	0.72	–	–	–
12.01–12.99 Injuries and accidents (external causes of death)	0.44	0.46	1.36	1.22	1.79	–	0.99	–
10.06 Congenital malformation	0.22	0.23	0.27	0.30	–	1.13	0.33	0.47
01.99 Other and unspecified infect diseases	–	–	–	–	–	–	0.33	–
06.01 Acute abdomen	–	–	0.27	–	0.72	3.17	1.96	3.32
01.08, 10.05 Tetanus	–	–	–	–	–	0.45	–	–
99 Indeterminate	0.67	1.40	1.90	1.52	1.43	4.08	1.31	1.42

WHO=World Health Organization.

Table 3 presents CSMF for the child (1–4 years) age group. Causes of death due to malaria declined from 35.81% in 2003 to 25.93% in 2007, followed by an increase to 46.00% in 2008 through 2009, then a decline to 44.10% in 2010. During the study period, acute respiratory infection including pneumonia decreased from 29.05 to 8.33%. A similar pattern was observed for HIV/AIDS-related deaths except in the years 2006–2007 and 2009.

**Table 3:** Cause-specific mortality fractions for children (1–4 years old), by year

	Child (1–4 year)							
	
	Calendar year							
	
	2003	2004	2005	2006	2007	2008	2009	2010
Number of deaths	444	485	388	409	270	537	392	288
WHO 2012 cause of death code								
01.05 Malaria	35.81	41.03	34.28	26.65	25.93	46.00	47.19	44.10
01.02 Acute respiratory infection, including pneumonia	29.05	17.94	20.10	19.56	19.26	11.55	6.38	8.33
01.03 HIV/AIDS-related death	24.32	24.33	22.94	29.83	33.70	20.30	31.63	24.65
01.04 Diarrheal diseases	2.48	7.63	5.67	8.07	6.30	6.33	4.34	6.60
01.06 Measles	1.80	1.24	3.61	2.20	1.85	2.23	2.04	0.35
03.02 Severe malnutrition	2.03	2.89	2.58	4.89	2.96	2.42	1.53	5.90
04.03 Sickle cell with crisis	0.90	0.82	2.84	1.96	1.48	1.30	0.26	0.35
12.05, 12.06 Injuries and accidents	0.68	1.24	2.59	3.18	1.48	1.31	1.80	2.43
03.01 Severe anemia	0.45	0.41	0.52	0.24	1.11	–	–	–
01.10 Pertussis	0.23	0.21	0.26	0.49	–	–	–	0.35
01.09 Pulmonary tuberculosis	0.23	–	–	–	–	0.74	–	0.35
01.01 Sepsis (non-obstetric)	–	0.62	0.26	–	0.74	–	–	–
06.01 Acute abdomen	0.23	–	0	0.24	1.11	4.28	3.06	3.12
01.07 Meningitis and encephalitis	–	–	0.77	–	–	0.56	–	1.04
06.02 Liver cirrhosis	–	–	–	–	–	0.74	–	0.35
08.01 Epilepsy	–	–	–	0.24	–	–	–	–
10.06 Congenital malformation	–	–	0.26	–	–	–	–	–
01.99 Other and unspecified infect diseases	–	–	–	–	–	0.19	0.26	1.04
07.01 Renal failure	–	–	–	–	–	0.19	0.51	–
99 Indeterminate	1.80	1.65	3.35	2.44	4.07	1.86	0.77	1.04

WHO=World Health Organization.

## Discussion

This study assessed cause-specific childhood mortality fractions in the KEMRI/CDC HDSS using the InterVA-4 model. The model is easy to use, requires a very short turnaround time, and is less expensive for reviewing VA questionnaires for ascertaining cause of death since it is an automated computer algorithm (21) that does not require the use of clinicians. Additionally, the model gives as many as three possible causes of death and the likelihood of occurrence of each cause as compared to clinicians’ review. Currently, the InterVA model may be the only freely available computer-based automated method for interpreting cause of death at the population level using VA data (23).

Our study reported a 25.29% decline in the under-5 mortality rate during the first 5 years of the study period, which translated to an annual decrease of 14% (IRR= 0.86, 95% CI: 0.85, 0.88). This could be associated with the scale-up of interventions targeting children in the study area (27). A surprising and remarkable increase in childhood mortality reported in 2008 was followed by a steady decline through the end of the study period, reaching similar estimates to those observed in 2007. It is suggested that this dramatic change in mortality could be due to the disruption of essential services during the post-election violence late that year and stockouts of artemether–lumefantrine (AL) when no other effective antimalarial drug was available (27).

According to this InterVA analysis, birth asphyxia was the main cause of death among children younger than 4 weeks old living in the study area. The high burden of this condition may be due to unsafe deliveries since over 60% of deliveries within the study area occur at home (28) with the help of non-professional birth attendants. The decline in birth asphyxia in the last 4 years of the study period could be a result of an increase in the number of pregnant women attending antenatal care, and an increase in deliveries at health facilities and with the help of skilled attendants (22).

Pneumonia was the second overall largest cause of death in under-5 children and neonates, and it was the largest cause of death in the infant group. Pneumonia has been described elsewhere as a major cause of child mortality in Sub-Saharan Africa (3, 9), and this highlights the urgent need for pneumonia prevention through vaccination, reduction of indoor pollution, and early adequate treatment of pneumonia to reduce childhood mortality in poor or developing countries. The observed age pattern of pneumonia deaths, in which the infant age group had the highest burden, is consistent with morbidity data (data not shown) from a health facility in the study area (22). Yearly decline was observed in all age groups except in neonates during the study period. The steady decrease could be associated with improved vaccination coverage and care seeking (29), and the change to a more effective treatment of pneumonia (30).

Preterm birth is a major cause of death in neonates (31, 32). In our study, cause of death due to prematurity was the third largest cause in neonates, and this steadily increased over the study period. The pattern implies a weak health system, such as low coverage of skilled clinical care for maternal and child health.

Malaria was the leading cause of death in under-5 year olds during the study period and was also implicated as the major killer among children between 1 and 4 years old. In general, about one in four deaths of children aged 1–4 years was due to malaria infection. In the infant age group, it was the second most frequent cause of death. From 2004 to 2007, malaria cases (27) and mortality rapidly declined in the study area. This may be due to the use of more effective first-line malaria treatment drugs: amodiaquine in 2004 and atemether–lumefantrine in 2006 (27). A study on child mortality in the same area also showed a decrease in all-cause mortality (6, 27). This implies that the decrease in overall mortality could be due to a decline in malaria deaths, given the significant contribution of malaria deaths.

Significant increases in malaria and neonatal pneumonia mortality reported in the year 2008 and sustained through 2010 could be as a result of the disruption of essential services during the post-election violence from December 2007 to February 2008, and the ensuing stockouts of malaria drugs (27). However, the sustained increase in cause-specific mortality is a matter for further investigations.

We observed an increase in HIV/AIDS-related deaths in children aged 1–4 years in the first 5 years of the study period. Surprisingly, during the same period, prevention of mother-to-child transmission of HIV uptake increased (33), which should have resulted in fewer HIV infections and hence lower mortality due to HIV/AIDS.

Diarrheal-related deaths were also one of the main contributors to child mortality, accounting for 8% of infant deaths. Hospital-based diarrheal mortality studies in African children have reported mortality due to acute diarrhea ranging from 1.9 to 37% of all deaths in the Gambia and Nigeria, respectively, with most of these deaths occurring within the first year of life (34). A study on the risk factors among children younger than 5 years old who were hospitalized with diarrhea in the study area also reported a case fatality rate of 9.3% (35). Globally, mortality due to diarrhea has been decreasing (3). Surprisingly, this was not the case in our study. Yearly increases of diarrheal mortality were reported in infants over the study period except in 2009, yet in-home water treatment was promoted in the community during the same period (27).

Malnutrition did not feature as one of the leading causes of death in our study, yet it is considered one of the main causes of childhood deaths in Sub-Saharan Africa (3, 31, 36). This finding is surprising because a similar study using clinician review in ascertaining cause of death from the same VA data (27) placed malnutrition as the third major cause of death in under-5 children. The low reporting of this condition could be due to the poor sensitivity of the InterVA-4 model in distinguishing between malnutrition and HIV/AIDS because the two conditions share a number of common symptoms such as weight loss and chronic diarrhea. Clinician CSMFs for HIV/AIDS were slightly lower (27) compared to the one obtained from the model.

Although our study reported a decline in the childhood all-cause mortality rate during the study period, this was not mirrored in all of the CSMFs. For instance, increases in diarrheal diseases and severe malnutrition as causes of death were reported in the child age group, although these increases were not significant. A significant increase of sepsis cases was also observed in the neonatal age group.

Our results were largely comparable with previously reported causes of death based on the clinician reviews, hospital deaths, and health facility sick visits within the study area (5, 27). Among the under-5 children, InterVA and clinician interpretations suggest malaria, acute respiratory infections including pneumonia, HIV/AIDS-related deaths, and diarrhea as major causes of death in childhood.

Routine mortality data collected in health and demographic systems play an important role in assessing child survival patterns. Understanding population-based cause-specific mortality in resource-constrained settings, where the majority of childhood deaths occur outside of a health facility, requires a reliable (reproducible) technique to be used in cause-of-death determination.

## Supplementary Material

Childhood cause-specific mortality in rural Western Kenya: application of the InterVA-4 model
