# Supplementary material for: Childhood cause-specific mortality in rural Western Kenya: application of the InterVA-4 model
Source: Glob Health Action. 2014 Oct 29;7:10.3402/gha.v7.25581. doi: 10.3402/gha.v7.25581 (PMC4221497; doi:10.3402/gha.v7.25581)
Supplement: Childhood cause-specific mortality in rural Western Kenya: application of the InterVA-4 model [file GHA-7-25881-s001.pdf]

Supplementary table:

Table 1: Cause-specific mortality fractions for under-five children (0-4 years)

	Under-five children (0-4 years)							
	Calendar year							
	**2003**	**2004**	**2005**	**2006**	**2007**	**2008**	**2009**	**2010**
Number of deaths	979	997	821	804	608	1098	780	534
**WHO 2012 cause of death code**								
01.01 Sepsis (non-obstetric)	-	0.50	0.24	-	0.66	0.09	0.13	-
01.02 Acute respiratory infection, including pneumonia	41.06	32.50	32.03	30.85	30.43	19.31	16.67	15.36
01.03 HIV/AIDS related death	16.65	14.94	16.20	19.90	21.71	13.30	20.64	18.73
01.04 Diarrheal diseases	2.76	6.72	6.70	8.83	7.73	7.83	5013	7.49
01.05 Malaria	25.03	30.29	26.19	21.14	19.41	35.61	37.69	38.20
01.06 Measles	1.53	2.11	2.68	2.36	1.64	1.73	2.05	0.94
01.07 Meningitis and encephalitis	0.20	0.20	0.37	0.25	0.33	1.18	0.77	2.43
01.08, 10.05 Tetanus	-	-	-	-	-	0.18	-	-
01.09 Pulmonary tuberculosis	0.10	-	-	-	-	0.36	-	0.19
01.10 Pertussis	0.20	0.10	0.12	0.25	-	-	-	0.19
01.99 Other and unspecified infect diseases	-	-	-	-	-	-	0.26	0.56
03.01 Severe anaemia	0.20	0.20	0.24	0.12	0.49	-	-	-
03.02 Severe malnutrition	1.12	1.40	1.22	2.86	1.48	1.46	0.90	3.75
04.03 Sickle cell with crisis	0.51	0.40	1.46	1.00	1.48	0.91	0.38	0.19
06.01 Acute abdomen	0.10	-	0.12	0.12	0.82	3.37	2.31	3.00
06.02 Liver cirrhosis	-	-	-	-	-	0.36	-	0.19
07.01 Renal failure	-	-	-	-	-	0.09	0.26	-
08.01 Epilepsy	0.20	0.10	-	0.12	0.33	-	-	-
10.01 Prematurity	0.10	0.10	0.37	0.50	0.16	0.46	0.90	0.75
10.02 Birth asphyxia	3.27	3.61	2.68	3.36	4.61	3.28	2.69	0.75
10.03 Neonatal pneumonia	3.68	2.81	2.68	2.36	1.97	2.28	3.08	2.81
10.04 Neonatal sepsis	0.41	0.40	0.37	0.37	0.66	1.46	1.03	0.94
10.06 Congenital malformation	0.10	-	0.24	0.12	-	0.73	0.26	0.37
10.99 Other and unspecified neonatal Causes of death	1.12	1.20	1.71	1.37	2.14	2.28	1.79	0.56
12.01 Road traffic accident	-	0.20	-	-	-	-	-	0.19
12.03 Accident fall	-	-	-	-	-	0.18	0.13	
12.04 Accid drowning and submersion	-	0.20	0.24	0.75	0.49	0.27	0.38	0.56
12.05 Accident exposure to smoke, fire & flame	0.31	0.20	0.61	1.12	0.82	0.09	0.51	0.37
12.06 Contact with venomous plant/animal	0.10	-	0.49	-	-	-	0.26	0.19
12.06 Accid poisoning and noxious subs	0.10		0.24	-	-	0.09	-	-
12.09 Assault	-	-	0.26	0.25		-	-	-
12.01-12.99 Injuries and accidents (external causes of death	-	-	0.12	-	-	-	-	-
12.10 Exposure to force of nature		0.20	0.12	-	-	-	-	-
12.99 Other and unspecified external CoD	-	-	-	-	0.16	-	-	-
99 Indeterminate	1.12	1.50	2.56	1.87	2.47	3.01	1.67	1.31
